# Medical Charlatans

**Published:** 1893-02

**Authors:** 


### Medical Charlatans.

In the introduction to his work on physiology, Prof. John William Draper very plainly states that the medical profession has itself to blame for having charlatans, and for the necessity of ethical codes. Dr. Draper's exact words are: "Who ever heard of sects among watch-makers, or quacks among engineers? . . . In medicine, the quack only exists because there is doubt. . . . The practice of medicine must rest on an exact anatomy, and a sound physiology. As soon as it is brought to this, empiricism will disappear of itself; it will need no legal enactments, no ethical codes for its destruction."—*Post-Graduate.*

